# Comprehensive Simulations for Ultraviolet Lithography Process of Thick SU-8 Photoresist

**DOI:** 10.3390/mi9070341

**Published:** 2018-07-05

**Authors:** Zai-Fa Zhou, Qing-An Huang

**Affiliations:** Key Laboratory of MEMS of the Ministry of Education, Southeast University, Nanjing 210096, China; zfzhou@seu.edu.cn

**Keywords:** SU-8 photoresist, cellular automaton, lithography simulation, modeling, fast marching

## Abstract

Thick SU-8 photoresist has been a popular photoresist material to fabricate various mechanical, biological, and chemical devices for many years. The accuracy and precision of the ultraviolet (UV) lithography process of thick SU-8 depend on key parameters in the set-up, the material properties of the SU-8 resist, and the thickness of the resist structure. As feature sizes get smaller and pattern complexity increases, accurate control and efficient optimization of the lithography process are significantly expected. Numerical simulations can be employed to improve understanding and process design of the SU-8 lithography, thereby allowing rapid related product and process development. A typical comprehensive lithography of UV lithography of thick SU-8 includes aerial image simulation, exposure simulation, post-exposure bake (PEB) simulation, and development simulation, and this article presents an overview of the essential aspects in the comprehensive simulation. At first, models for the lithography process of the SU-8 are discussed. Then, main algorithms for etching surface evolvement, including the string, ray tracing, cellular automaton, and fast marching algorithms, are introduced and compared with each other in terms of performance. After that, some simulation results of the UV lithography process of the SU-8 are presented, demonstrating the promising potential and efficiency of the simulation technology. Finally, a prospect is discussed for some open questions in three-dimensional (3D) comprehensive simulation of the UV lithography of the SU-8.

## 1. Introduction

SU-8 is a negative, epoxy-type, ultraviolet (UV) photoresist based on EPON SU-8 epoxy resin from Shell Chemical, The Hague, The Netherlands, that has been originally developed, and patented (US Patent No. 4882245 (1989)), by International Business Machines Corporation (IBM, Armonk, NY, USA) [1]. The first SU-8 products were reported by MicroChem Inc., (previously Microlithography Chemical Corp., Westborough, MA, USA) [2]. Another company, Gersteltec Sarl, Pully, Switzerland [3], has also bought a license from IBM to produce and now sells the SU-8. Anyway, the products from the two companies are similar. Because thick SU-8 has excellent chemical stability, resistance against solvents, mechanical properties, and optical properties, it becomes a popular photoresist material for the fabrication of many microelectromechanical systems (MEMS) structures and devices [4,5,6,7,8,9,10,11,12,13,14,15,16,17,18], including optical pick-up head [5], microlens [12], microfluidic channels [13], micropump [15], microneedles [17], cantilevers [18], and so on. Compared with X-ray LIGA (Lithografie, Galvanoformung, Abformung) [16] based on SU-8 using a very expensive synchrotron source [6,7], UV lithography of the SU-8 utilizes an inexpensive UV light source. This makes the UV lithography of the SU-8 more accessible. Currently, different UV lithography of SU-8, such as vertical lithography, inclined (tilted) lithography, multidirectional lithography, interference lithography, and so on, are widely used for fabricating MEMS structures and devices. It should be mentioned that there are several commercially available types of the same negative photoresist, for example TMMR/TMMF from Tokyo Ohka Kogyo Co., Ltd., Kawasaki, Japan [19,20]. The reviewed simulation approaches in this paper can also be extended to simulate the lithography process of these, if corresponding model parameters are extracted.

There are many process parameters that will affect the final lithography profiles, such as the gap between the mask and SU-8 layer, exposure dose, mask shape and development time, and so on. So it becomes complicated and time-consuming to accurately control the dimensions of microstructures and to optimize the lithography processes by using conventional repeated-experiment method. Simulations are useful to deal with these problems [21,22,23,24,25,26,27], for the efficiency in understanding the fundamental effects of individual process parameters. In integrated circuits (IC) fields, lithography simulators have been playing an indispensable role in process optimization and design of micron, sub-micron, and nanometer devices for many years, and some simulation software tools have been commercialized. However, lithography process simulations of thick photoresist such as SU-8 cannot be accurately implemented by the software specified for thin photoresist, because there are some differences between these two lithography processes [28,29], such as nonlinear factors, the special exposure method for thick SU-8 lithography process, and so on. To overcome this problem, some specified researches for the UV lithography simulations of thick photoresists have been reported during the past several years [30,31,32,33,34,35,36,37,38,39,40,41,42,43,44,45,46,47,48].

This article presents an overview of the essential aspects in the comprehensive simulation of UV lithography processes of SU-8. In Section 2, the basic mechanisms of UV lithography process of SU-8 and various models are introduced and discussed. In Section 3, main algorithms for the etching surface advancement simulation, including the string, ray tracing, cellular automata, and fast marching algorithms, are presented and compared. In Section 4, simulations of the UV lithography processes of SU-8 are presented and discussed. Finally, a prospect is given for some open questions in three-dimensional (3D) comprehensive simulations of the UV lithography processes of SU-8.

## 2. Simulation Models for Ultraviolet (UV) Lithography Process of Thick SU-8 Photoresist

### 2.1. Basic Simulation Process

**Figure 1:**
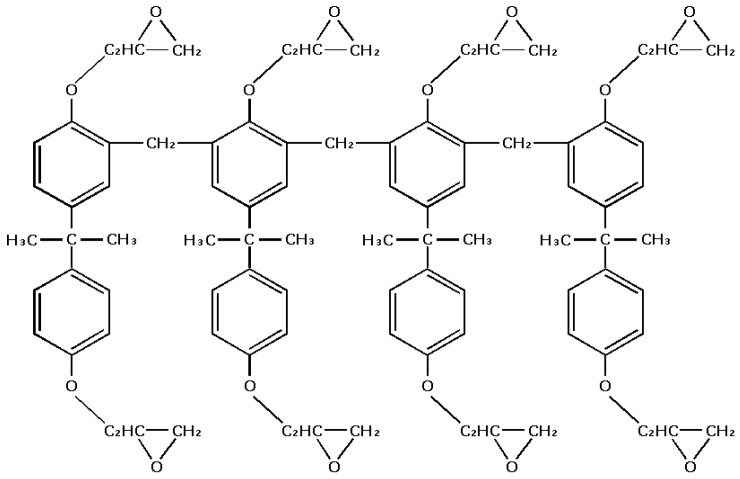
SU-8 molecule with epoxy groups.

The original SU-8 series consists of three basic components: an epoxy called EPON SU-8 resin (Miller-Stephenson Chemical Company, Danbury, CT, USA), a solvent called gamma-butyrolactone (GBL), and a photoacid generator from the family of the triarylsulfonium salts [1,2,3]. Figure 1 shows the structure of an SU-8 molecule, containing eight epoxy groups. In actual fact, the molecules have a wide variety of sizes and shapes. Currently, the original SU-8 resist series (SU-8 5, SU-8 50, SU-8 100, etc.) and its newer formulations (SU-8 2000 series and SU-8 3000 series) are available. These newer formulations may offer improved adhesion on some substrates versus the original formulation. For example, SU-8 3000 series resists use cyclopentanone for the primary solvent and can be spun into films ranging from 2 µm to 75 µm in a single coat. Although the contents for various SU-8 are a little different, the general sequence of lithography steps is basically the same as the following [49,50,51]: substrate pretreatment, spin-coating of SU-8, soft bake, exposure, post-exposure bake (PEB), development, rinse, and hardbake. Currently, lithography simulations of the SU-8 are studied by constructing separate models for exposure, PEB, and development processes, similar to other thin negative chemical amplification photoresists in the IC fabrication field [52,53,54]. For the evaluation of effects from some lithography steps such as substrate pretreatment, spin-coating, soft bake, rinse and dry, an experimental characterization method is used, because the models for these lithography steps have not been implemented efficiently. However, they are still no corresponding simulation models for some phenomenon during the lithography process, such as shrinkage behavior [55].

**Figure 2:**
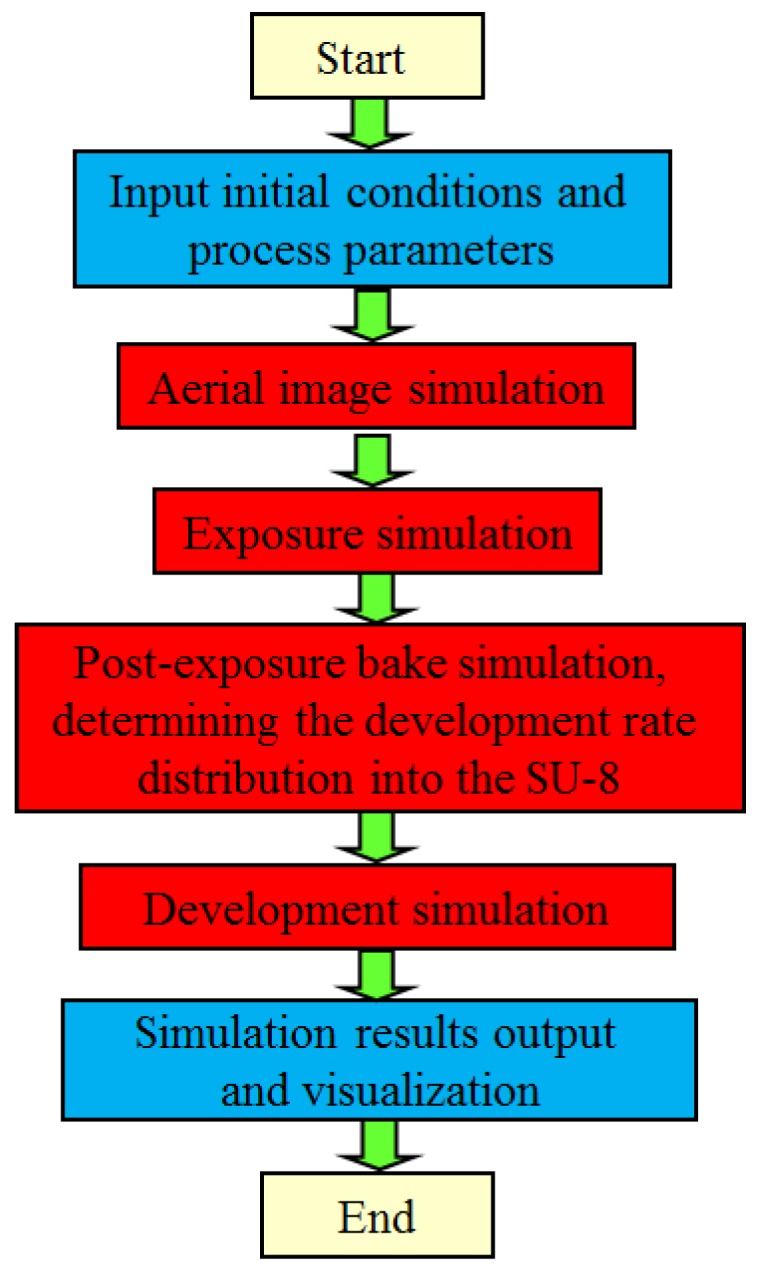
Simulation flow chart for the ultraviolet (UV) lithography of SU-8.

As shown in Figure 2, the aerial image simulation, exposure simulation, PEB simulation, and development simulation determine the final simulation profiles of the SU-8 for a typical comprehensive lithography simulation. For the beginning, the initial conditions and process parameters, including mask data, exposure time, UV light intensity, SU-8 layer thickness, UV light incident angle, gap between mask and SU-8 layer, reflectivity of substrate, PEB time and temperature for both two steps, development time and temperature, and so on, should be input into the simulation system. Then, the illumination of the mask on top of the SU-8 by the incident UV light source is obtained by aerial image simulation. During the following exposure simulation, the UV light propagation, as well as the generation of the photoacid HSbF_6_ within the SU-8, is simulated. After that, the post-exposure bake (PEB) simulation is used to describe the photoacid-catalyzed crosslinking reaction, obtaining the cross-linked site concentration in the SU-8 [56,57]. Thus, the development (etching) rate distribution into the SU-8 can be obtained according to the obtained cross-linked site concentration. Finally, with the development rate distribution, the development profiles of the SU-8 can be obtained by the appropriate etching surface evolvement algorithms introduced in Section 3. The final development profile data can be visualized and output for further analysis for various purposes. Generally speaking, the aerial image simulation and development simulation are time-consuming steps.

### 2.2. Aerial Image Simulation Models

Although the optical projection exposure method has been the main method for many years, contact and proximity exposure methods of the SU-8 using a so-called mask aligner are still the cost efficient exposure method in the current MEMS field [58,59]. The ultimate goal of aerial image simulation is to obtain the light intensity distribution in the SU-8. Because the wavelength of the 365 nm UV light is rather long, optical diffraction from masks will affect the precision of the lithography pattern. Furthermore, the reflection and refraction at the interface between the air gap (or compensation materials) and the SU-8, and the SU-8 and the substrate will also affect the precision of the lithography pattern [36,40,47]. Currently, aerial image simulations are based on two main categories of models. One category is based on scalar diffractive theory [29,30,31,38,41,47,59] and the other is based on rigorous electromagnetic field theory [60,61,62,63,64,65,66,67]. The former can be further divided into models based on the Fresnel diffraction [27,28,29,30,31,59], Fresnel–Kirchhoff diffraction [38,40,41,47], diffractive angular spectrum theory [33], and so on. While the latter can be further divided into models based on the finite difference time domain (FDTD) method [60,61,62] and waveguide method [63,64,65,66,67].

Compared with the models based on rigorous electromagnetic field theory [58,59,60,61,62,63,64,65], including the waveguide method, models based on the scalar diffractive theory are significantly faster (usually over hundreds of times or even more). Theoretically, these models are only valid for mask features that are large and thin compared with the wavelength of exposure light. This is not a problem for the UV lithography of the SU-8, which always satisfies this condition. The limitation of these models comes from the simulation accuracy, the application capability for special lithography methods such as inclined lithography, and the numerical costs for large-scale 3D simulations. To solve these problems, many studies have been conducted during the past several years. Tian et al. presented a correction to the Fresnel diffraction theory to increase the accuracy, depending on the scalar diffraction theory of light propagation [31]. For accurate and rapid simulation, incorporating the effect of reflection, transmission on the boundary in the oblique incidence case, and diffraction in the photoresist, Tang et al. extend the angular spectrum theory [68] to the light intensity distribution calculation for the lithography of thick photoresists. The propagation of the incident UV light will be refracted on the compensation material/SU-8 interface for the inclined UV lithography, and it is difficult to deal with the refraction in the Fresnel–Kirchhoff diffraction integral equation. To deal with this issue, Zhu et al. presented the “mask shifting approach” to handle the diffraction and refraction effects simultaneously [40]. To further reduce the light intensity distribution calculation time, Zhou et al. presented a method using an adaptable element size in *x*, *y*, and *z* directions for the whole calculation domain [47]. Using this method, the calculation time for 3D simulation can be reduced from 2 h to about 16 min for a moderately complex mask shape. 

**Figure 3:**
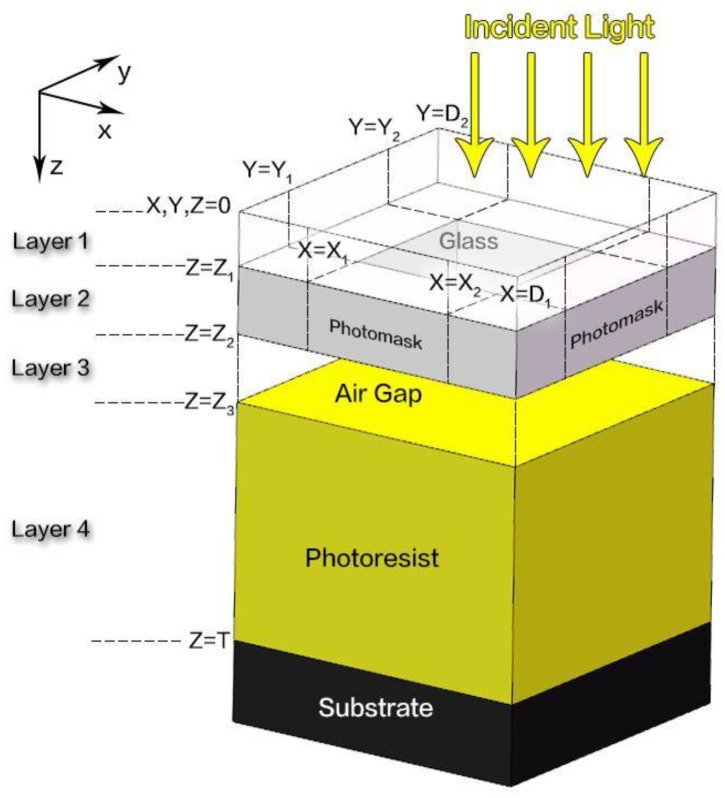
Three-dimensional schematic of the waveguide method.

Compared with models based on scalar diffraction theory, the models based on rigorous electromagnetic field theory are more accurate. Since the pioneering work from Wong et al. [60], FDTD becomes an attractive method in rigorous mask diffraction simulation. FDTD uses a space domain discretization of the electric and magnetic field on equidistant staggered grids, solving the object fields at the mask rigorously. All diffraction, refraction, interference, absorption, and polarization effects are calculated out in the near field of the mask without approximation. FDTD can further reduce memory requirements and time per simulation by incorporating a graded mesh. The idea of the waveguide method is based on the theory of the Fourier modal method, and it is well adapted to lithography simulation with many typical mask geometries. The first prototype of the waveguide method was presented by Nyyssonen [63], and later this method was extended to a three-dimensional domain [64]. The concept of the waveguide method is the same. At first, the simulated structure is sliced into several layers with homogeneous optical properties along the vertical direction [29], *z*-direction, as shown in Figure 3. Then, the Fourier series is employed for the material parameters and electromagnetic field in all layers. Simultaneous equations formed by these expanded series and Maxwell Equations give an eigenvalue problem. After that, a hybrid approach using vector potentials is applied to couple electromagnetic fields in each layer. With extensive speed and convergence optimizations, an acceptable performance for this case is realized. Finally, using proper boundary conditions, the eigenvalue problem is solved, giving the light intensity distribution information throughout the propagation region. 

The combination of the waveguide method with domain decomposition techniques [66] and parallelized simulations enables the efficient and rigorous diffraction simulation for larger mask areas of the thin photoresist lithography [67]. The simulation time is less than 30 min on a 3.2 GHz Dual Core Pentium PC for a 245λ × 245λ × 6λ (λ is the wavelength of the exposure light) simulation areas. This means that a simulation speed over 15 times faster than that of the FDTD has been achieved. Theoretically, the models based on rigorous electromagnetic field theory can accurately simulate light intensity distribution in SU-8, if the limitations from the computation cost can be neglected. With the development of computer technology and the parallelization methods based on graphics processing units (GPUs), these limitations would seem to be partly solved in the near future. 

### 2.3. Exposure Simulation Models

**Figure 4:**
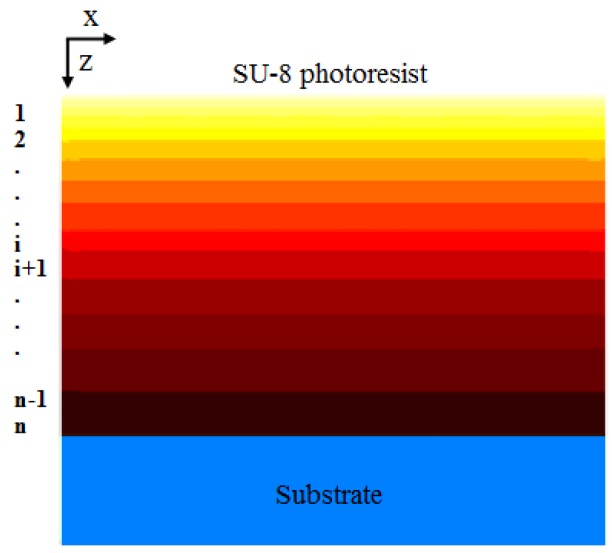
Schematic diagram of the concentration distribution of contents in thick SU-8 photoresist.

During the exposure process, the photoreaction initiator decomposes and a strong acid (HSbF_6_) is generated within the SU-8. According to the UV light intensity distribution into the SU-8, exposure models are used to describe the kinetics in the SU-8 during this process. The original *Dill* model [21], presented by *Dill* and his team at IBM for the mathematical equations describing basic lithography processes, has played an important role in conventional optical lithography simulations of thin photoresists for many years [68]. However, some special cases should be concerned for thick SU-8 compared with thin photoresists, such as the nonlinear factors in the exposure process of thick photoresists like SU-8. For example, the concentration distribution of contents and the refractive index vary along *z* direction, as shown in Figure 4. To solve this problem from the nonlinear characteristics, the original *Dill* model should be modified [33,42,69,70].

Liu et al. presented an enhanced *Dill* model using a set of modeling parameters, taking into account of the effects caused by light diffraction or the scattering in the AZ4562 thick photoresist (MicroChemicals GmbH, Ulm, Germany) and the change of photoresist refractive index in the exposure process [71]. Similarly, Zhou et al. developed an improved *Dill* model describing the nonlinear effects in the SU-8 photoresist [45], to simulate the exposure kinetics during the exposure process by the following [38,47]:

(1) $$\frac{\partial m(x,y,z,t)}{\partial t}=-I(x,y,z,t)m(x,y,z,t)C(z)$$

(2) $$\alpha_{i}(x,y,z,t)=A(z)m(x,y,z,t)+B(z)$$

(3) $$I(x,y,z,t)=I_{0}(x,y,z)\cdot \exp(-\int_{0}^{l}\alpha_{i}(x,y,z,t)dz)$$

(4) $$C_{A}(x,y,z,t)=1-m(x,y,z,t)$$

where the SU-8 was divided into *n* layers, and *α_i_* was the absorption coefficient for the *i*th layer. *I*_0_(*x*, *y*, *z*) was the light intensity. *m*(*x*, *y*, *z, t*) and *C_A_*(*x*, *y*, *z, t*) denoted the normalized photoacid generator concentration and HSbF_6_ concentration, respectively. *z*(μm) was a variable to indicate the photoresist depth (the distance from top to bottom of the SU-8 layer). *l* was the distance that the UV light passes through in the SU-8. The *Dill* parameters varied with the SU-8 thickness:

(5) $$A(z)=i_{1}+i_{2}\;z+i_{3}\;z^{2}$$

(6) $$B(z)=j_{1}+j_{2}\;z+j_{3}\;z^{2}$$

(7) $$C(z)=l_{1}+l_{2}\;z+l_{3}\;z^{2}$$

By fitting experimental results using the conventional “Dill graphical method” [72], the *Dill* parameters for various SU-8 thickness can be separately obtained, then *i*_1_, *i*_2_, *i*_3_, *j*_1_, *j*_2_, *j*_3_, *l*_1_, *l*_2_, and *l*_3_ are fitted out. The *Dill* graphical method involves extracting the *A* and *B* parameters from the starting and ending sample transmittance values, while the *C* parameter is estimated from the slope of the initial portion of the transmittance curve. Based on this principle, some specific exposure parameter measurement tools have been available for many years, such as the ABC analyzer manufactured by Lithotech, Saitama, Japan [32,73,74]. For the SU-8 2000 series, the values for these constants were fitted to be as follows: *i*_1_ = −0.0031, *i*_2_ = 4.7878 × 10^−6^, *i*_3_ = −3.0860 × 10^−9^, *j*_1_ = 0.0079, *j*_2_ = 7.5510 × 10^6^, *j*_3_ = 4.0971 × 10^9^, *l*_1_ = 0.0865, *l*_2_ = −1.3078 × 10^−4^, and *l*_3_ = 6.1193 × 10^−8^ [38].

### 2.4. Post-Exposure Bake (PEB) Simulation Models

For the PEB process, HSbF_6_ catalyzes ring-openings of epoxies and turns them into cross-linked sites. Thus, the SU-8 resin will transfer from a low-molecular-weight material to a highly cross-linked three-dimensional polymer network, and the degree of cross-linkage of SU-8 depends on the PEB temperature. The polymer network is significantly less soluble than the resin without cross-linking reaction. Bobbitt [75] introduces the details of catalytic chemical reactions of epoxy. Studies reveal that the physical properties, such as Young’s modulus and the coefficient of thermal expansion, highly depend on UV exposure and PEB conditions [76], and these kinds of variations in physical properties have been investigated using molecular dynamics simulations in different groups [77,78]. However, this simulation approach can not currently be incorporated into the lithography profile simulations discussed in this paper. 

The chemical reactions and diffusion of HSbF_6_ happen simultaneously and couple with each other during the PEB process, because the HSbF_6_ concentration is not uniform in SU-8. Thus, the coupled reaction-diffusion kinetics must be considered simultaneously. As for thin photoresists in the IC field, some complicated and accurate PEB models have been presented [24,25,62]. However, if they are expected to be used for the PEB process of the SU-8 lithography, many efforts should be put into the measurement of various model parameters. Currently, the model originally developed by Zuniga et al. [79] can be used to describe the PEB process for the SU-8. As the reaction order for HSbF_6_ and cross-linked sites is 1 for the SU-8 lithography, the equations for the PEB process of the SU-8 can be expressed by the following [2,38,47]:

(8) $$\frac{\partial C_{cs}}{\partial t}=k_{1}(1-C_{cs})C_{A}^{q}$$

(9) $$\frac{\partial C_{A}}{\partial t}=\nabla \cdot (D_{acid}\nabla C_{A})-k_{2}C_{A}$$

where Equation (8) describes the reaction leading to the de-protection of the polymer sites, and Equation (9) describes the HSbF_6_ evolution in time and space during the PEB process. *C_cs_* was the normalized concentration of the cross-linked sites where epoxy rings become open, and one cross-linked site is created when one epoxide ring is open. *q* was the order of the crosslinking reaction, *D_acid_* was the diffusivity of HSbF_6_, *k*_1_ is the site-crosslinking reaction coefficient, and *k*_2_ is the photoacid loss reaction coefficient. *k*_1_, *k*_2_, and *D_acid_* had an Arrhenius relationship with temperature. The coefficients in Equations (8) and (9) can be determined experimentally, referring to the reported detailed procedures and equipments such as the PAGA-100 deprotection reaction analysis system (manufactured by Lithotech, Saitama, Japan [74]) for chemical amplification resists [19,80,81]. For example, Sensu et al. determined the parameter values of the PEB model for SU-8 photoresists using experimental methods by PAGA-100 [30]. 

### 2.5. Development Simulation Models

The first step to simulate the development of the SU-8 in developer is to calculate the dissolution (development) rate of each point in the SU-8. Various dissolution rate models have been proposed to describe the relationship between dissolution rate distribution within the SU-8 and cross-linked site concentration, such as the *Mack* model [82,83], *Weiss* model [84], *Notch* model [85,86], and *Enhanced Notch* model [85,86]. For example, the *Notch* Model is expressed as follows:

(10) $$R_{Notch}(C_{cs})=R_{\max}{\left(1-C_{cs}\right)}^{n_{1}}\left[\frac{(a+1){\left(1-C_{cs}\right)}^{n\_notch}}{a+{\left(1-C_{cs}\right)}^{n\_notch}}\right]+R_{\min}$$

(11) $$a=\frac{\left(n\_notch+1\right)}{\left(n\_notch-1\right)}{\left(1-C_{csth\_notch}\right)}^{n\_notch}$$

where *R*_min_ and *R*_max_ are the minimum and maximum dissolution rate of the SU-8 photoresists, respectively; *n*_1_ denotes the steepness of the rate curve; *M_th-notch_* is the threshold value of *C_cs_* where the notch occurs; and *n_n-notch_* is the measure for the strength of the notch. As shown by Equations (10) and (11), the parameter values for former mentioned models are dependent on the PEB conditions.

The development parameters in the above-mentioned models can be determined using specific equipment, such as RDA-790 by Lithotech, Saitama, Japan [74], according to the reported procedures [30,87]. It should also be mentioned that some other parameters, besides the cross-linked site concentration, should also be measured for the above models. As we know, agitation methods are usually adopted for high aspect ratio structures during the development process, and the dissolution rate will be increased over several times, compared with the development process without an agitation method [2,38,47]. For the SU-8 development simulation, Sensu et al. provided some *Mack* model parameters for SU-8 3000 series [58]: *R*_max_ = 240.98 nm/s, *R*_min_ = 0.08 nm/s, *n_n-notch_ =* 19.86, and *M_th-notch_* = 0.33 for the development process without agitation methods. Zhou et al. provided some *Notch* model parameters for SU-8 2000 series [37]: *R*_max_ = 1.02 μm/s, *R*_min_ = 0.00009 μm/s, *n*_1_ = 1.2, *n_n-notch_ =* 20.0, and *M_th-notch_ =* 0.43 for the development process with agitation. Currently, the *Weiss* model parameters and *Enhanced Notch* model parameters are still not available for the SU-8. Furthermore, the dissolution rate of the SU-8 is depth-dependent [38,88], and the swelling effect will also affect the size of pattern edges and sidewall profiles of the SU-8 [38,42]. Although no accurate physical models for these effects are available, it is necessary to adopt the depth-dependent dissolution rate effect, and the swelling model, to improve the simulation accuracy for some cases [38,41,88]. We notice that the swelling effect seems more significant for the original SU-8 resist series than for its newer formulations (SU-8 2000 series and SU-8 3000 series) [2,44]. With the dissolution rate distribution in the SU-8, the propagation of the development profiles can be simulated using etching surface evolvement algorithms introduced in the Section 3. 

## 3. Algorithms for Etching Surface Evolvement Simulation

To capture the evolution of the lithography profiles of SU-8, the development (etching) surface should be first represented by any one of etching surface evolvement algorithms (techniques). Thus, the etching surface evolvement with time can be simulated based on the dissolution rate distribution into the SU-8. Etching surface evolvement algorithms are one of the key components for profile simulations, as they must accurately reflect the evolvement without excessive computation. Currently, there are string algorithms [89,90,91,92,93,94], ray-tracing algorithms [95,96,97], cellular automata algorithms [98,99,100,101,102,103,104,105,106,107,108,109,110], and fast marching algorithms [111,112,113,114,115,116,117,118,119,120,121] that have been reported for the etching surface evolvement simulation of lithography simulations. Table 1 summarizes brief feature comparisons of these algorithms.

### 3.1. String Algorithms

**Figure 5:**
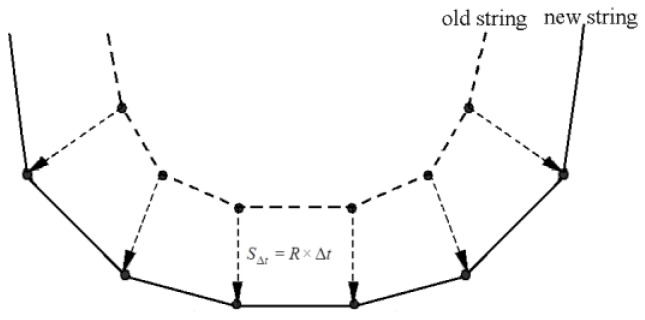
Schematic diagram of string algorithm.

The string method was introduced by Jewett et al. [89] and was initially used to describe photoresist development for thin photoresists. The surface is represented by a string of nodes connected by straight line segments, as shown in Figure 5. The etching surface is advanced in small discrete time steps (Δt) by moving the etching point along a vector normal to the local surface. The advancement distance is the average of the advancement length of two adjacent segments. The string algorithms require little memory and computation time, as they only keep to track the etching surface. Jewett et al. [89] compared the string algorithm with the cell-based and ray-tracing algorithms using a test case of electron-beam exposure. They demonstrate that the string algorithm is more accurate and computationally more efficient than the cellular automata algorithms. The main disadvantage of the string algorithms is that loops will form in the boundary surface, especially when the etching rate in the material has a highly varied spatial distribution. The limited step size should be kept small to prevent loop formation and ensure accuracy of the solution. Segments must be removed when their length becomes comparable to the step size, otherwise loops can form. Loops form at the intersections of converging segments, which in turn results in physically incorrect surface evolution [94]. The de-looping algorithms must also be incorporated to ensure accuracy and correctness accuracy. Especially for the case of 3D simulations, the straight-line segments must be changed into triangles and polygons for representing the etching surface. Thus, the de-looping in 3D becomes much more computationally expensive, as it involves all triangle or polygon intersections in a mesh containing thousands of triangles or polygons.

### 3.2. Ray Tracing Algorithms

A ray tracing algorithm was an application of the differential ray equation, and began to be used for etching surface advancement during lithography simulations in the 1970s [95]. In the ray tracing algorithms, the radial or directional vectors are initialized perpendicularly to the initial surface, and then each point moves along the direction vector in the discrete minute interval. After each step, the direction vector of the point is recalculated by the discrete ray equation. The developed and undeveloped regions are defined with different development rates. Actually, the propagation of the etching vectors is calculated like the propagation of light using Snell’s law of refraction. The index of refraction of the SU-8 is defined as $n_{1}=R_{\max}^{\prime }/R(x,y,z)$, where $R_{\max}^{\prime }$ is the maximum value of the local development rate *R*(*x*, *y*, *z*). The evolvement of the etching front can be implemented based on the etching vectors. 

Because the ray trajectory is only determined by the previous trajectory and the corresponding etching rate at that point, there is no need to track the relationship between different rays. Theoretically speaking, the ray tracing algorithm is easy to implement. However, the actual situation is a little more complicated. The ray tracing algorithm does not describe the whole etching surface, but only the points on the surface. In order to reconstruct the etched morphology from the end point of the trajectory, a high density point is needed in a certain area. Even so, it is not easy to reconstruct the etching surface by the ray ends, because the rays are independent of each other. Furthermore, the added rays must be in the sparse area of the ray, and sometimes some areas will not be etched because of inappropriate selection of the initial rays. The difficulties in reconstruction of the surface from independent rays make the ray tracing algorithms not very attractive for 3D etching simulations.

### 3.3. Cellular Automata Algorithms

The cellular automata algorithms were first presented by John von Neumann following Ulam’s suggestions [98]. The computation domain is discretized into a set of square cells for 2D cases or cubic cells for 3D cases that are associated with different materials. The states at each cell are involved simultaneously based on the previous states of the cell and its neighboring cells at discrete time steps, according to a set of “local rules” [99,102,103,104,105,106,107,108,109,110]. Because the “local rule” is only local relationships among neighboring cells, the governing equation for the whole computation domain is not necessary. Surface evolvement is represented by the removal or addition of cells or some parts of cells. For many years, the cellular automata algorithms have been used to simulate the shallow trench isolation etch with chlorine plasma [99,100], plasma etching of SiO_2_ [101], anisotropic etching of silicon [101,102,103,104], photoresist development [105,106,107,108,109], and so on. A great advantage of the cellular automata algorithms is the ease of handling topological changes for arbitrary geometries and the easy with which they are implemented in 3D. 

The preferential etching in different directions is great in the cellular automata algorithm with von Neumann neighborhood, significantly reducing the simulation accuracy compared with Moore neighborhood. For simplification, during the following part of this paper, discussions focus on the cellular automata algorithm with Moore neighborhood. For the development simulation, the SU-8 layer is divided into a matrix of identical square cells [106] or cubic cells [107,109] with side length *a.* Here, 3D cases are discussed as an example. There are 6 adjacent cells, 12 diagonal cells, and 8 point cells in the neighborhood of a target cell. A cell is gradually etched by the etchant flowing from its neighbors. The local state of a cell is defined as the ratio of the etched volume to the total volume. 

The 3D static cellular automaton algorithm is slow and inefficient [107], because the simulation program must process entire cells, even those lying in the interior of the photoresist, leading to reduction of the simulation speed and increase of the computer memory usage. For a simulation matrix of *n* × *n* × *n,* the computation time goes roughly as *o*(*n*^4^), where *n* is the number of cells in one dimension. At the same time, the 3D static cellular automata algorithm needs to store the state array of the previous time step and the present time step (8*n*^3^ or 16*n*^3^ bytes). Furthermore, 4*n*^3^ bytes are also required to store the etching rate array. Thus, the memory storage is larger than 12*n*^3^ bytes. To improve simulation performance, Zhou et al. presented a 3D dynamic cellular automaton algorithm, using a dynamic memory allocation scheme [109], only processing the boundary cells. For the same simulation matrix of *n* × *n* × *n*, the computation time goes roughly as *o*(*n*^3^), while the memory usage is about 6.4*n*^3^ (2.4*n*^3^ bytes to store the cell state flag array and the boundary cell array and additional 4*n*^3^ bytes to store the etching rate array and the time compensation values). Detailed results, using a test function, revealed that for a simulation matrix of 100 × 100 × 100, the speed of the 3D dynamic cellular automaton algorithm is increased by over 10 times compared with that of the 3D static cellular automaton algorithm, while the memory usage is reduced by about 50% [109]. This makes the 3D dynamic cellular automaton algorithm practical for some 3D lithography simulation cases. Even so, the simulation speed is still a serious problem, limiting the application extension of the 3D lithography simulations of the SU-8. Here, a set of data is presented to illustrate this problem. For a simulation matrix with 500 × 500 × 500 using the 3D dynamical cellular automaton algorithm, the typical simulation time usually approaches several hours for a personal computer configuration: OS Windows XP SP3, CPU Intel Core2@2 GHz, DRAM 2 GB. 

### 3.4. Fast Marching Algorithms

The fast marching algorithm, originally introduced by Sethian [111,112], briefly cited as fast marching method (FMM), is an optimally efficient algorithm for solving problems of front evolution where the front speed is monotonic. Namely, it is a stationary version of the general level set method [113,114]. The FMM is a powerful numerical technique for analyzing and computing moving fronts, and it has been applied to a wide variety of problems including seismic wave propagation, photoresist development, medical imaging, and optimal path planning. The flow of FMM can be divided into two parts: initialization and marching forward. In the initialization, the object is meshed into grids. During the marching forward to capture the etching surface evolvement, the FMM computes the time *T*_(*x*, *y*, *z*)_ when the SU-8-developer interface passes through each point in a grid in the simulation area. The dissolution t rate of the SU-8, $R_{\left(x,y,z\right)}^{\prime }$, is only a function of position, and the Eikonal equation $\left|\nabla T_{\left(x,y,z\right)}\right|R_{\left(x,Y,Z\right)}^{\prime }=1$ must be satisfied. Because the SU-8-developer interface can only advance in one direction during the whole photoresist dissolution progress, an “upwind” scheme for viscosity solutions of Hamilton Jacobi equations [115] can be adopted to solve the Eikonal equation. 

Although the original FMM is accurate, fast, stable, and easy to implement in three dimensions, some research efforts have been made to further improve the original FMM by decreasing the time complexity and raising the method’s precision. Yatziv et al. used a calendar untidy priority queue to reduce the time complexity [120]. Hassouna et al. adopted a multi-stencil to increase the precision [121]. From available results, the simulation speed of current fast marching algorithms was above eight times faster than the 3D dynamical cellular automaton algorithm [109]. However, original 3D FMM needs too many memory elements during simulations. For example, for a simulation matrix of *n* × *n* × *n*, the computation time goes roughly as 0(C*n^3^log(*n*)) + 0(*n*^2^), where *C* is a pretty small constant and *n* is the number of grids in one dimension [109]. For memory elements, the original 3D FMM needs 12.4*n*^3^ bytes of memory in total, including 4*n*^3^ bytes to store the time values, 4*n*^3^ bytes to store the dissolution rate array, 0.1 × 4*n*^3^ bytes to store the min-heap structure of narrowband points, and 4*n*^3^ bytes for companion array to point out the index of nodes in the min-heap. The simulation using the original FMM is faster than the 3D dynamical cellular automata algorithm, but needs too many memory elements. For the simulation matrix of 500 × 500 × 500 and the same personal computer configuration (OS Windows XP SP3, CPU Intel Core2@2 GHz, DRAM 2 GB) as in Section 3.3, the simulation cannot be implemented, as the memory limit of the computer is exceeded. To overcome this problem, Zhou et al. developed a 3D fast marching method based on full hash table (full hash fast marching method, briefly cited as HFMM) to reduce the memory usage [42]. 

**Figure 6:**
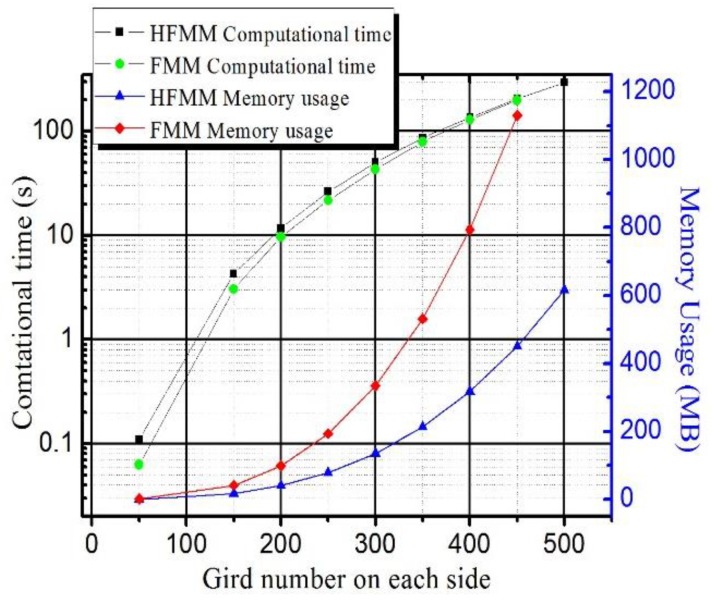
Comparisons of the running time and memory usage of the original fast marching method (FMM) and the full hash fast marching method (HFMM) on different grid sizes using a typical dissolution rate function *F* = 2((*x* − 0.5)^2^ + (*y* − 0.5)^2^).

As the original FMM only calculates nodes within the narrowband, a min-heap structure is developed to store the time values of the narrow band nodes. At the same time, a fast sweeping method is developed to search the node with the minimum time value in the narrow band, and to update the min-heap structure. Furthermore, a hash table is developed to store all data for the nodes in the narrow band, so the required memory elements are reduced. To illustrate the performance of the 3D HFMM, Figure 6 shows the running time and memory usage for a typical test function. Figure 6 indicates that the HFMM and the original FMM have nearly the same computational speed. However, nearly 60% of the computer memory elements in HFMM can be saved without any computational speed and accuracy loss, compared with the original FMM. With these advantages and the development of computer technology, algorithms extended from FMM will become increasingly competitive for the lithography simulation, especially for large-scale 3D lithography simulations of thick photoresists like SU-8. 

## 4. Simulations and Discussions

**Figure 7:**
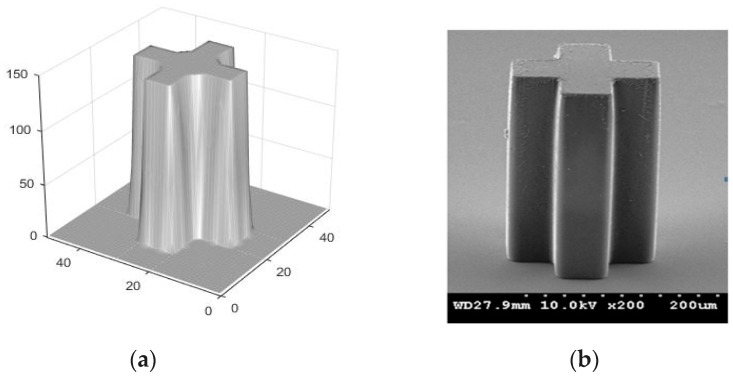
Three-dimensional lithography profiles of a cross-shaped mask with perpendicular incidence using the binary threshold approach, with light intensity obtained by using waveguide method. (**a**) Simulation result; (**b**) experimental result: exposure time = 250 s, development time = 12 min. The first step PEB condition was 65 °C/12 min and the second step PEB condition was 95 °C/40 min.

**Figure 8:**
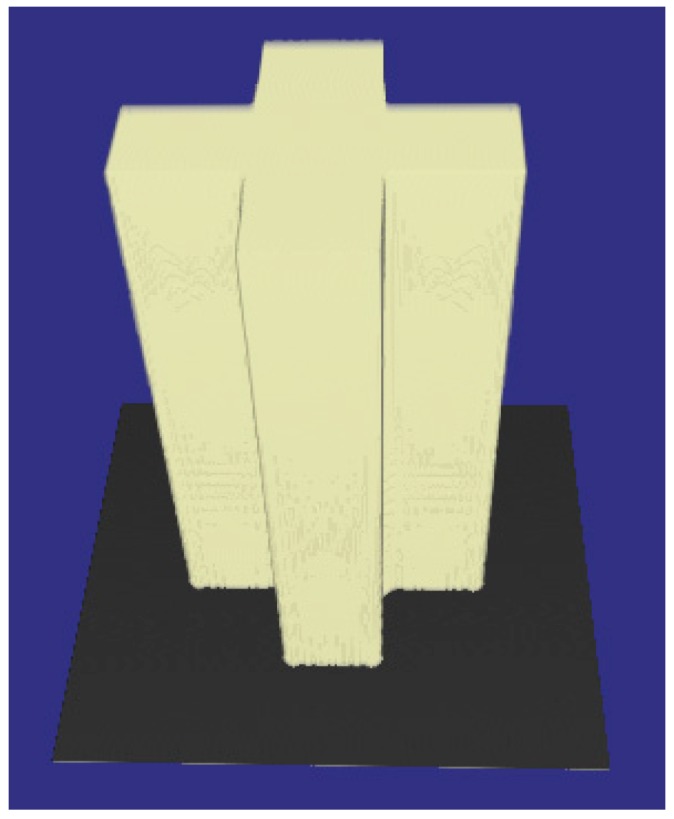
Three-dimensional lithography profiles of a cross-shaped mask with perpendicular incidence by using a comprehensive lithography simulation: exposure time = 250 s, development time = 12 min. The first step PEB condition was 65 °C/12 min and the second step PEB condition was 95 °C/40 min.

Based on the models mentioned above and the algorithms for etching surface evolvement simulation, comprehensive simulation systems of the UV lithography process of SU-8 can be implemented. However, different level simulations of UV lithography, besides comprehensive simulations including all lithography steps or models, have been put into applications for various purposes and have achieved some success in many cases. For example, Cheng et al. [29] estimated the wall profiles and resolution for the near-field lithography of thick photoresist based on the Fresnel diffraction theory and exposure kinetics model. Chuang et al. [30] established an aerial image model to analyze the sidewall profiles of UV exposure of SU-8, leading to a novel method to reduce the diffraction problem from air gap using glycerol as a compensator to bridge out the air gap between the mask and SU-8. This method can greatly improve the sidewall straightness of high-aspect ratio SU-8 microstructures. Based on the effects of the Fresnel diffraction and absorption, Kang et al. [36] further presented an efficient model to predict the sidewall profiles of SU-8. Two exposure methods avoiding negatively sloped sidewall profiles were developed by utilizing the model, and SU-8 performs without undercut for the replication of optical waveguides were achieved using the exposure methods. Miao et al. [44] thoroughly analyzed and discussed the influences of different process parameters on the final surface profiles of micro lenses fabricated using UV lithography of SU-8, combining simulations and experiments. Yang et al. [46] built a complete 3D Fresnel–Kirchhoff diffraction model to predict the microstructure profiles fabricated by backside lithography of SU-8, combining a binary threshold approach. With the guidance of the above simulations, researchers can predict and optimize the lithography profiles [29,30], propose and evaluate some novel lithography techniques, and develop novel MEMS devices. However, it should be acknowledged that efficient comprehensive simulations are more suitable for understanding the fundamental effects of individual lithography step parameters and for optimizing the UV lithography process of SU-8. The reason lies in the fact that many lithography parameters will affect the final lithography profiles, including UV light distributions, exposure energy, development time, and so on. The accuracy of the simulation results is limited if very few parameters can be considered at the same time. For example, Figure 7 shows the simulation and experimental results for the lithography profiles of a cross-shaped mask with perpendicular incidence under UV source with 365 nm (2.6 mW/cm^2^) radiation using the binary threshold approach. Here, the normalized light intensity of 0.33 is defined as the threshold value of SU-8. The maximum error between the simulation and experimental profiles in Figure 7 is about 10.4%. While the maximum error between the simulation profile of a comprehensive simulation process (Figure 8) and the corresponding experimental profile (Figure 7b) is about 7.6%. From this comparison, the disadvantage of the binary threshold approach in accuracy is evident.

**Figure 9:**
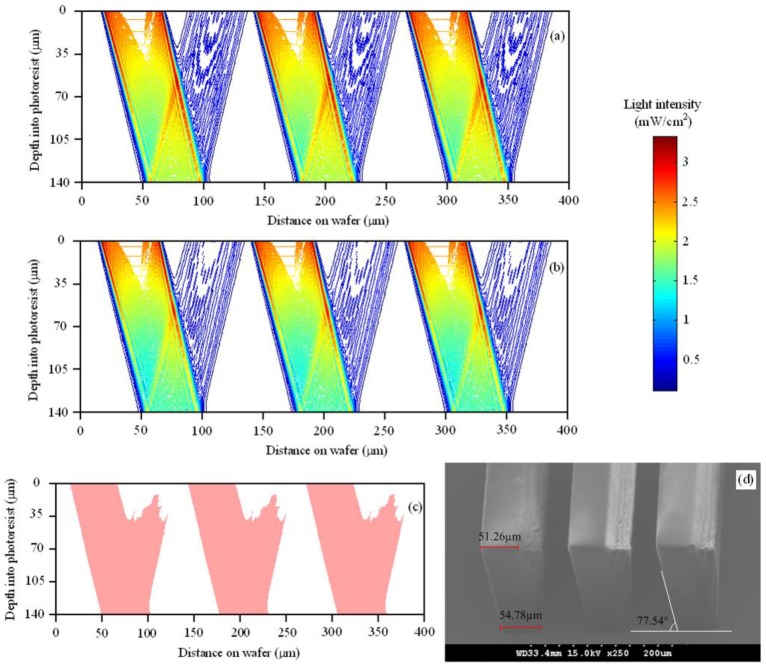
Simulation and experimental results of the inclined lithography of the SU-8 for 23.5° UV incident angle with reflected UV on bare silicon wafer: exposure time = 240 s, development time = 12 min, line/space = 50/75 μm. (**a**) Predicted light intensity distribution at the beginning of the exposure process; (**b**) predicted light intensity distribution at the end of the exposure process; (**c**) simulation result; and (**d**) experimental result. Reproduced with permission from [44].

**Figure 10:**
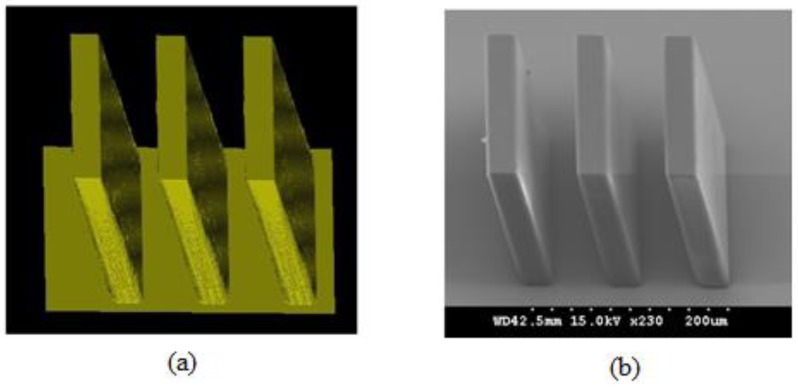
(**a**) Simulation result and (**b**) experimental result of the inclined UV lithography for 140 ± 12 μm thick SU-8 with a “|||” shape mask for 30° UV incident angle with TiO_2_ film as antireflection layer: exposure time = 240 s, development time = 12 min, line/space = 30/60 μm, the first step PEB condition was 65 °C/12 min, and the second step PEB condition was 95 °C/35 min. Reproduced with permission from [47].

Several research groups have reported their efforts to develop comprehensive simulation systems and to use the simulation systems for device design and process optimization. Zhou et al. have been developing comprehensive 2D and 3D simulation systems for UV lithography of the SU-8 for several years [37,41,44,47], and a series of 2D and 3D simulations have be implemented for vertical, inclined, and multi-directional lithography methods. Figure 9 shows the 2D simulation and experimental results of the inclined UV lithography of the SU-8 for 23.5° UV incident angle on bare silicon wafer [44]. Figure 9a,b reveal that the light absorption coefficient of the SU-8 is not a constant during the whole exposure processes. The reason lies in the fact that the cross-linked polymer has a higher absorption coefficient than the SU-8 before cross-linking reaction. The results also reveal that if only the UV light distribution is considered, the accuracy will be inevitably reduce. As both the exposed and unexposed SU-8 absorb material, the reflected UV light will be absorbed when passing through the SU-8. Thus, the energy of the reflected UV is relatively low to initiate cross-linking reactions of all SU-8, especially the SU-8 on the top, so the reflection effect is more notable at the bottoms of the SU-8 microstructures, as shown in Figure 9c,d. As more factors on the development profiles can be considered in the comprehensive simulation system [44], the simulation accuracy has been increased, compared with the profiles obtained using aerial image simulation and the threshold approach [40]. The largest line width variation between the experimental and simulation profiles in Figure 9c,d is less than 1.72 μm. Figure 10 shows the 3D simulation and experimental profiles of inclined UV lithography for 30.0° incident angle, with TiO_2_ film as an antireflection layer. The simulation time is about 39 min for the whole lithography process (an impressive high simulation speed), because adaptable element size and HFMM are adopted [47]. The maximum error between the simulation and experimental profiles in Figure 10 is less than 7.3%. With the simulation system, Zhou et al. have investigated the effects of different lithography parameters on the line width and topography deviation for lots of SU-8 microstructures, including exposure time, development time, line/space, and mask shapes [39,47]. 

Compared with current lithography simulation tools of thin photoresists in the IC field, the applications of UV lithography simulations of the SU-8 or other thick photoresists are significantly limited. The lithography simulations are now critical and standard tools for IC fabrication, thin photoresist design, and its evaluation. To some degree, UV lithography simulations of the SU-8 are behind of the lithography technology development of the SU-8. Up to now, few simulation tools are available. IntelliSense, a famous MEMS CAD company (Nanjing, China), released a simulation module *Exposure* for the UV lithography of SU-8 by collection with Southeast University, China [122].

**Figure 11:**
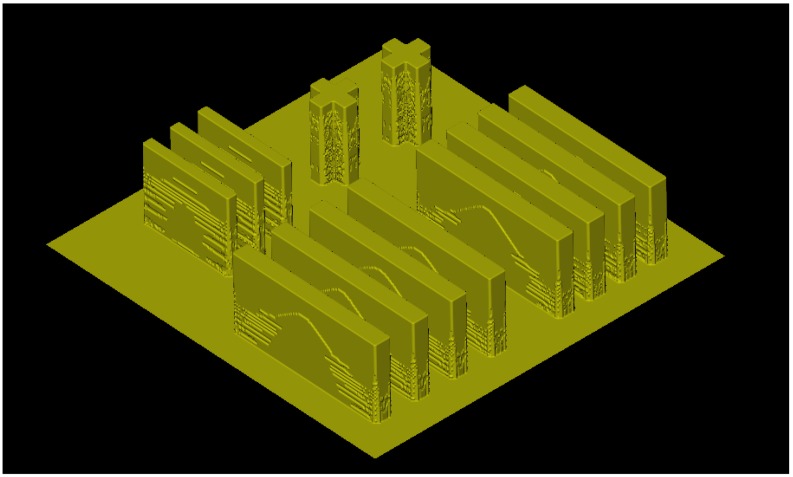
3D simulation results of UV lithography for 240 ± 12 μm thick SU-8 with a complex mask shape on glass wafer with TiO_2_ film as antireflection layer: exposure time = 380 s, development time = 13 min, the first step PEB condition was 65 °C/15 min, and the second step PEB condition was 95 °C/45 min.

To extend the application of lithography simulations of the SU-8, several issues should be concerned. From the view of practical application, 3D simulations for various masks, as shown in Figure 11, even full chip level simulations, are greatly expected for the lithography process optimization and dimensional control of microstructures. However, these simulations require too many computer resources, and the computer resources for certain PCs are certainly limited. To deal with this problem, fast and efficient algorithms should be developed. Furthermore, current main stream algorithms, such as cellular automaton and fast marching algorithms, have an intrinsic parallel updating nature, and the corresponding simulations are highly inefficient when performed on classical central processing units (CPUs). Thus, improved parallel methods for the UV lithography simulation of the SU-8 on graphics processing units (GPUs) will be helpful. The recent cost-efficient examples of the parallel architectures for silicon anisotropic etching simulations based on GPU [123,124] provide a useful reference for this kind of research. Similarly, former mentioned image simulations based on rigorous electromagnetic field are highly flexible and accurate, and deal relatively easily with various problems in lithography simulation. However, the computer calculation ability limits their applications in UV lithography simulation of the SU-8. The usage of GPUs is also a possible solution to this problem using massively parallel computing approaches. In addition, an important aspect for a lithography simulator is accuracy. More accurate (if possible) physically-based models for various lithography simulation steps are expected over a wider range of process conditions. Finally, more efforts are expected to extract and optimize more model parameters. Research efforts along these lines will make the simulation technology more practical and this can satisfy the needs of UV lithography technology development of the SU-8.

## 5. Conclusions

The essential aspects in the comprehensive simulation of UV lithography of the SU-8 are reviewed and discussed. This shows that great progress has been made in this field during the past several years. Available lithography models for exposure, PEB, and development steps are analyzed, and the main algorithms for the etching surface evolvement simulation are reviewed with their advantages and challenges. Some typical simulations of the UV lithography of the SU-8 are also introduced and discussed. Finally, several challenging issues regarding the simulation speed and accuracy are discussed, to realize further extension of the applications of the UV lithography simulations of the SU-8.

## Figures and Tables

**Table 1:** Feature comparisons of different etching surface advancement algorithms for the ultraviolet (UV) lithography simulation of the SU-8.

Algorithm		Main Merits	Main Disadvantages	References
String algorithm		Very fast, accurate, less memory elements	Easy to form loops, unstable, not easy to be extended to 3D simulations	[89,90,91,92,93,94]
Ray tracing algorithm		Fast, accurate, less memory usage	Unstable, unsuitable for 3D simulations	[95,96,97]
Cellular automata (CA) algorithm	Static CA algorithm (with Moore neighborhood)	Very stable, easy to be extended to 3D simulations	Very slow, not very accurate, much memory usage	[106,107]
	Dynamic CA algorithm (with von Neumann neighborhood)	Stable, easy to be extended to 3D simulations	Slow, not accurate (preferential etching)	[105,110]
	Dynamic CA algorithm (with Moore neighborhood)	Accurate, stable, easy to be extended to 3D simulations, less memory usage	Relatively slow	[38,108]
Fast marching algorithm	Original fast marching algorithm	Fast, accurate, very stable, easy to be extended to 3D simulations	Much memory usage	[111,112,115]
	Full hash fast marching algorithm	Fast, accurate, very stable, less memory usage, easy to be extended to 3D simulations	Need additional over 120 lines of computer program codes, compared with the original fast marching algorithm	[43,47]

## References

[1] Shaw J.M., Gelorme J.D., LaBianca N.C., Conley W.E., Holmes S.J. (1997). Negative photoresists for optical lithography. IBM J. Res. Dev..

[2] MicroChem Negative Epoxy Resists. http://www.microchem.com.

[3] Swiss Made SU-8 Photoepoxy Functional Products. http://www.gersteltec.ch.

[4] Han M., Lee W., Lee S.K., Lee S.S. (2004). 3D microfabrication with inclined/rotated UV lithography. Sens. Actuators A.

[5] Hung K.Y., Hu H.T., Tseng F.G. (2004). Application of 3D glycerol-compensated inclined-exposure technology to an integrated optical pick-up head. J. Micromech. Microeng..

[6] Balslev S., Romanato F. (2005). Functionalized SU-8 patterned with X-ray lithography. J. Vac. Sci. Technol. B.

[7] Becnel C., Desta Y., Kelly K. (2005). Ultra-deep X-ray lithography of densely packed SU-8 features: II. Process performance as a function of dose, feature height and post exposure bake temperature. J. Micromech. Microeng..

[8] Yoon Y.K., Park J.H., Allen M.G. (2006). Multidirectional UV lithography for complex 3-D MEMS structures. J. Microelectromech. Syst..

[9] Sato H., Yagyu D., Ito S., Shoji S. (2006). Improved inclined multi-lithography using water as exposure medium and its 3D mixing microchannel application. Sens. Actuators A.

[10] Campo A., Greiner C. (2007). SU-8: A photoresist for high-aspect-ratio and 3D submicron lithography. J. Micromech. Microeng..

[11] Hung K.Y., Liang T.H. (2008). Application of inclined-exposure and thick film process for high aspect-ratio micro structures on polymer optic devices. Microsyst. Technol..

[12] Cheng Y.W., Chiang T.H., Ngoc D.L., Do D.B., Hsu C.C. (2009). Fabrication of microlens arrays based on the mass transport effect of SU-8 photoresist using a multi-exposure two-beam interference technique. Appl. Opt..

[13] Colin D.J., Calame J.P., Morag G. (2010). UV-LIGA microfabrication of 220 GHz sheet beam amplifier gratings with SU-8 photoresists. J. Micromech. Microeng..

[14] Moser Y., Forti R., Lehnert J.S. (2011). Suspended SU-8 structures for monolithic microfluidic channels. Microfluid. Nanofluid..

[15] Rouabah H.A., Park B.Y., Zaouk R.B., Morgan H., Madou M.J. (2011). Design and fabrication of an AC-electro-osmosis micropump with 3D high-aspect-ratio electrodes using only SU-8. J. Micromech. Microeng..

[16] Becker E.W., Ehrfeld W., Hagmann P., Maner A., Münchmeyer D. (1996). Fabrication of microstructures with high aspect ratios and great structural heights by synchrotron radiation lithography, galvanoforming, and plastic moulding (LIGA process). J. Microelectron. Eng..

[17] Stavrinidis G., Michelakis K., Kontomitrou V., Giannakakis G., Sevrisarianos M. (2016). SU-8 microneedles based dry electrodes for electroencephalogram. Microelectron. Eng..

[18] Martinez V., Behr P., Drechsler U., Polesel M.J., Potthoff E., Voros J., Zambelli T. (2016). SU-8 hollow cantilevers for AFM cell adhesion studies. J. Micromech. Microeng..

[19] Hirai Y., Sugano K., Tsuchiya T., Tabata O. (2010). Embedded microstructure fabrication using developer-permeability of semi-cross-linked negative resist. J. Microelectromech. Syst..

[20] Photo-Patternable Permanent Materials for Structure Fabrication. http://microfluidics.tok.co.jp/.

[21] Dill F.H., Neureuther A.R., Tuttle J.A. (1975). Modeling projection printing of positive photoresists. IEEE Trans. Electron Devices.

[22] Erdmann A., Fühner T., Evanschitzky P., Agudelo V., Freund C., Michalak P., Xu D.B. (2015). Optical and EUV projection lithography: A computational view. Microelectron. Eng..

[23] Henke W., Weiss M., Schwalm R., Pelka J. (1990). Simulation of proximity printing. Microelectron. Eng..

[24] Neureuther A.R., Mack C.A. (1997). Handbook of Microlithography, Micromaching, and Microfabrication.

[25] Cole D.C., Barouch E., Conrad E.D., Yeung M. (2001). Using advanced simulation to aid microlithography development. Proc. IEEE.

[26] Karafyllidis I., Haguel P.I., Thanailakis A., Neureuther A.R. (2000). An efficient photoresist development simulator based on cellular automata with experimental verification. IEEE Trans. Semicond. Manuf..

[27] Zhu Z.R., Swecker A.L., Strojwas A.J. (2004). METRO-3D: An efficient three-dimensional wafer inspection simulator for next-generation lithography. IEEE Trans. Semicond. Manuf..

[28] Cheng Y., Lin C.Y., Wei D.H., Loechel B., Gruetzner G. (1999). Wall profile of thick photoresist generated via contact printing. J. Microelectromech. Syst..

[29] Wang F. (2015). Aerial Image Simulation for Optical Lithography Process. Master’s Thesis.

[30] Chuang Y.J., Tseng F.G., Lin W.K. (2002). Reduction of diffraction effect of UV exposure on SU-8 negative thick photoresist by air gap elimination. Microsyst. Technol..

[31] Tian X., Liu G., Tian Y., Zhang P., Zhang X. (2005). Simulation of deep UV lithography with SU-8 resist by using 365 nm light source. Microsyst. Technol..

[32] Sensu Y., Sekiguchi A., Mori S. (2005). Profiles simulation of SU-8 thick film resist. J. Photopolym. Sci. Technol..

[33] Yang R., Wang W.J. (2005). A numerical and experimental study on gap compensation and wavelength selection in UV-lithography of ultra-high aspect ratio SU-8 microstructures. Sens. Actuators B.

[34] Tang X.G., Gao F.H., Guo Y.K. (2005). Analysis and simulation of diffractive imaging field in thick film photoresist by using angular spectrum theory. Opt. Commun..

[35] Rumpf R.C., Johnson E.G. (2005). Comprehensive modeling of near-field nanopatterning. Opt. Express.

[36] Kang W.J., Rabe E., Kopetzet S. (2006). Exposure methods based on reflection and refraction effects in the field of SU-8 lithography. J. Micromech. Microeng..

[37] Zhou Z.F., Huang Q.A., Li W.H., Feng M., Lu W. (2007). Improvement of the 2D dynamic CA method for photoresist etching simulation and its application to deep UV lithography simulations of SU-8 photoresists. J. Micromech. Microeng..

[38] Zhou Z.F., Huang Q.A., Li W.H. The swelling effects during the development processes of deep UV lithography of SU-8 photoresists: Theoretical study, simulation and verification. Proceedings of the 6th IEEE Sensors.

[39] Tang X.G., Yang X.Y., Gao F.H., Guo Y. (2007). Simulation and analysis for microstructure profile of optical lithography based on SU-8 thick resist. Microelecton. Eng..

[40] Zhu Z., Zhou Z.F., Huang Q.A., Li W.H. (2008). Modeling, simulation and experimental verification of inclined UV lithography for SU-8 negative thick photoresists. J. Micromech. Microeng..

[41] Zhou Z.F., Huang Q.A., Huang Q.A. (2017). Modeling and simulation of SU-8 thick photoresist lithography. Micro Electro Mechanical Systems. Micro/Nano Technologies.

[42] Zhou Z.F., Huang Q.A., Li W.H. Simulations, analysis and characterization of the development profiles for the thick SU-8 UV lithography process. Proceedings of the 9th IEEE Sensors.

[43] Shi L.L., Zhou Z.F., Li W.H. A modified 3D fast marching simulation for thick photoresists lithography. Proceedings of the 10th IEEE Sensors.

[44] Zhou Z.F., Zhu Z., Huang Q.A., Li W.H. (2011). An efficient simulation system for inclined UV lithography processes of thick SU-8 photoresists. IEEE Trans. Semicond. Manuf..

[45] Miao Z.Y. (2013). Modeling and Simulation of Surface Profile Formation Process of Microlenses and Their Application in Optical Interconnection Devices. Ph.D. Thesis.

[46] Yang W.C., Huang Y.S., Shew B.Y., Fu C.C. (2013). Study on diffraction effect and microstructure profile fabricated by one-step backside lithography. J. Micromech. Microeng..

[47] Zhou Z.F., Shi L.L., Zhang H., Li W.H. (2014). Large scale three-dimensional simulations for thick SU-8 lithography process based on a full hash fast marching method. Microelectron. Eng..

[48] Huang Y.T., Hsu W.Y. (2014). A simulation model on photoresist SU-8 thickness after development under partial exposure with reflection effect. Jpn. J. Appl. Phys..

[49] Ong B.H., Yuan X., Tao S., Tjin S.C. (2006). Photothermally enabled lithography for refractive-index modulation in SU-8 photoresist. Opt. Lett..

[50] Jiang G.M., Baig S., Wang M.R. (2012). Prism-assisted inclined UV lithography for 3D microstructure fabrication. J. Micromech. Microeng..

[51] Hirai Y., Inamoto Y., Sugano K., Tsuchiya T., Tabata O. (2007). Moving mask UV lithography for three-dimensional structuring. J. Micromech. Microeng..

[52] Karafyllidis I. (1997). Simulation of the negative chemical amplification deep-ultraviolet process in integrated circuit fabrication. Microelectron. Eng..

[53] Fuhner T., Schnattinger T., Ardelean G. Dr. (2007). LiTHO—A development and research lithography simulator. Proc. SPIE.

[54] Karafyllidis I., Hagouel P.I., Neureuther A.R. (1999). Negative resist profiles of close-spaced parallel and isolated lines: Experiment, modelling and simulation. Microelectron. Eng..

[55] Yin Z.H., Cheng E., Zou H.L. (2017). Numerical study on the shrinkage behavior of SU-8 patterns. Microsyst. Technol..

[56] Berry A.K., Graziano K.A., Bogan L.E. (1989). Polymers in Microlithography.

[57] Reichmanis E., Houlihan F.M., Nalamasu O. (1994). Polymers for Microelectronics.

[58] Synopsys Sentaurus Lithography Simulation Software. http://www.synopsys.com.

[59] Bourdillon A.J., Boothroyd C.B., Kong J.R., Vladimirsky Y. (2000). A critical condition in Fresnel diffraction used for ultra-high resolution lithographic printing. J. Phys. D Appl. Phys..

[60] Wong A.K., Neureuther A.R. (1994). Mask topography effects in projection printing of phase-shifting masks. IEEE Trans. Electron Devices.

[61] Erdmann A., Evanschitzky P., Fühner T. (2006). Rigorous mask modeling using waveguide and FDTD methods: An assessment for typical hyper NA imaging problems. Proc. SPIE.

[62] Erdmann A., Fuhner T., Shao F., Evanschitzky P. (2009). Lithography simulation: Modeling techniques and selected applications. Proc. SPIE.

[63] Nyyssonen D. (1982). The theory of optical edge detection and imaging of thick layers. J. Opt. Soc. Am..

[64] Lucas K., Tanabe H., Strojwas A.J. (1996). Efficient and rigorous three-dimensional model for optical lithography simulation. J. Opt. Soc. Am..

[65] Evanschitzky P., Erdmann A. (2005). Three dimensional EUV simulations: A new mask near field and imaging simulation system. Proc. SPIE.

[66] Schellenberg F.M., Adam K., Matteo J., Hesselink L. (2005). Electromagnetic phenomena in advanced photomasks. J. Vac. Sci. Technol..

[67] Shao F., Evanschitzky P., Reibold D., Erdmann A. (2008). Fast rigorous simulation of mask diffraction using the waveguide method with parallelized decomposition technique. Proc. SPIE.

[68] Born M., Wolf E. (1999). Principles of Optics.

[69] Mack C.A. (2005). 30 Years of Lithography Simulation. Proc. SPIE.

[70] Sohn D.S., Sohn Y.S., Bak H.J., Oh H.K. (2001). Analysis of the relation between exposure parameters and critical dimension by response surface model. Proc. SPIE.

[71] Liu S.J., Du J.L., Duan X. (2005). Enhanced Dill exposure model for thick photoresist lithography. Microelectron. Eng..

[72] Henderson C.L., Pancholi S.N., Chowdhury S.A. (1997). Photoresist characterization for lithography simulation Part 2: Exposure parameter measurements. Proc. SPIE.

[73] Sekiguchi A., Minami Y., Matsuzawa T., Takezawa T., Miyakawa H. (1995). Measuring system of A, B, C Photoresist parameters. Electron. Commun. Jpn..

[74] Product information. http://www.ltj.co.jp/.

[75] Bobbitt M.M. (2001). The Effect of Chemistry and Network Stucture on Morphological and Mechanical Properties of Diepoxide Precursors and Poly(hydroxyethers). Ph.D. Thesis.

[76] Chung S.W., Park S. (2013). Effects of temperature on mechanical properties of SU-8 photoresist material. J. Mech. Sci. Technol..

[77] Tam L.H., Lau D. (2014). A molecular dynamics investigation on the crosslinking and physical properties of epoxy-based materials. RSC Adv..

[78] Yagyu H., Hirai Y., Uesugi A., Makino Y., Sugano K., Tsuchiya T., Tabata O. (2012). Simulation of mechanical properties of epoxy-based chemically amplified resist by coarse-grained molecular dynamics. Polymer.

[79] Zuniga M., Wallraff G., Tomacruz E. (1993). Simulation of locally enhanced three-dimensional diffusion in chemically amplified resists. J. Vac. Sci. Technol..

[80] Yoshino H., Matsumoto H. (1992). Simulation of chemical amplification resists. Jpn. J. Appl. Phys..

[81] Yamamoto H., Kozawa T., Tagawa S., Iwai T., Onodera J. (2009). Dissolution kinetics and deprotection reaction in chemically amplified resists upon exposure to extreme ultraviolet radiation. Proc. SPIE.

[82] Mack C.A. (1985). PROLITH: A comprehensive lithography model. Proc. SPIE.

[83] Mack C.A. (1992). New kinetic model for resist dissolution. J. Electrochem. Soc..

[84] Weiss M., Binder H., Schwalm R. (1995). Modeling and simulation of chemically amplified DUV resist using the effective acid concept. Microelectron. Eng..

[85] Arthur G., Mack C.A., Eilbeck N. (1998). Analyzing the dissolution characteristics of deep UV chemically amplified photoresist. Microelectron. Eng..

[86] Mack A., Arthur G. (1998). Notch model for photoresist dissolution. Electrochem. Solid State Lett..

[87] Arthur G., Mack C.A. A new development model for lithography simulation. Proceedings of the Olin Microlithography Seminar.

[88] Zanghellini J., Achenbach S., El-Kholi A., Mohr J., Pantenburg F.J. (1998). New development strategies for high aspect ratio microstructures. Microsyst. Technol..

[89] Jewett R.E., Hagouel P.I., Neureuther A.R., Duzer T.V. (1977). Line-profile resist development simulation techniques. Polym. Eng. Sci..

[90] Hamaguchi S., Rossnagel S.M. (1995). Simulations of trench-filling profiles under ionized magnetron sputter metal deposition. J. Vac. Sci. Technol..

[91] Levinson J.A., Shaqfeh E.S.G., Balooch M., Hamza A.V. (2000). Ion-assisted etching and profile development of silicon in molecular and atomic chlorine. J. Vac. Sci. Technol..

[92] Leunissen L.H.A., Jonckheere R., Ronse K., Derksen G.B. (2003). Influence of gate patterning on line edge roughness. J. Vac. Sci. Technol..

[93] Vyvoda MA., Li M., Graves D.B., Lee H. (2000). Role of sidewall scattering in feature profile evolution during Cl_2_ and HBr plasma etching of silicon. J. Vac. Sci. Technol..

[94] Arnold J.C., Sawin H.H., Dalvie M., Hamaguchi S. (1994). Simulation of surface topography evolution during plasma etching by the method of characteristics. J. Vac. Sci. Technol..

[95] Hagouel P.I. (1976). X-ray Lithographic Fabrication of Blazed Diffraction Gratings. Ph.D. Thesis.

[96] Toh K.K.H., Neureuther A.R., Scheckler E.W. (1994). Algorithms for simulation of three-dimensional etching. IEEE Trans. Comput. Aided Des. Integr. Circuit Syst..

[97] Cooperberg D.J. (2002). Semiempirical profile simulation of aluminum etching in a Cl2/BCl3 plasma. J. Vac. Sci. Technol..

[98] Neumann J. (1966). Theory of Self-Reproducing Automata.

[99] Hoang J., Hsu C.C., Chang J.P. (2008). Feature profile evolution during shallow trench isolation etch in chlorine-based plasmas I Feature scale modeling. J. Vac. Sci. Technol..

[100] Hsu C.C., Marchack N., Martin R., Pham C. (2013). Feature profile evolution during shallow trench isolation etching in chlorine-based plasmas III the effect of oxygen addition. J. Vac. Sci. Technol..

[101] Stout P.J., Rauf S., Nagy A., Ventzek P.L.G. (2006). Modeling dual inlaid feature construction. J. Vac. Sci. Technol..

[102] Zhou Z.F., Huang Q.A., Li W.H., Deng W. (2007). A cellular automaton-based simulator for silicon anisotropic etching processes considering high index planes. J. Micromech. Microeng..

[103] Zhou Z.F., Huang Q.A., Li W.H. (2009). Modeling and simulations of anisotropic etching of silicon in alkaline solutions with experimental verification. J. Electrochem. Soc..

[104] Gosálvez M.A., Xing Y., Sato K. (2008). Analytical solution of the continuous cellular automaton for anisotropic etching. J. Microelectromech. Syst..

[105] Scheckler E.W., Tam N.N., Pfau A.K., Neureuther A.R. (1993). An efficient volume-removal algorithm for practical three-dimensional lithography simulation with experimental verification. IEEE Trans. Comput. Aided Des. Integr. Circuit Syst..

[106] Karafyllidis I., Thanailakis A. (1995). Simulation of two-dimensional photoresist etching process in integrated circuit fabrication using cellular automata. Model. Simul. Mater. Sci. Eng..

[107] Karafyllidis I. (1999). A Three-dimensional photoresist etching simulator for TCAD. Model. Simul. Mater. Sci. Eng..

[108] Zhou Z.F., Huang Q.A., Li W.H., Lu W. (2005). A novel 2-D dynamic cellular automata model for photoresist etching process simulation. J. Micromech. Microeng..

[109] Zhou Z.F., Huang Q.A., Li W.H., Lu W. (2007). A novel 3D dynamic cellular automata model for photoresist-etching process simulation. IEEE Trans. Comput. Aided Des. Integr. Circuit Syst..

[110] Strasser E., Selberherr S. (1995). Algorithms and models for cellular based topography simulation. IEEE Trans. Comput. Aided Des. Integr. Circuit Syst..

[111] Sethian J.A. (1996). Fast marching level-set methods for three-dimensional photolithography development. Proc. SPIE.

[112] Sethian J.A. (1995). A fast marching level set method for monotonically advancing fronts. Proc. Natl. Acad. Sci. USA.

[113] Osher S., Sethian J.A. (1988). Fronts propagating with curvature dependent speed: Algorithms based on the Hamilton-Jacobi formulation. J. Comput. Phys..

[114] Adalsteinsson D., Sethian J.A. (1995). A level set approach to a unified model for etching, deposition, and lithography II: Three-dimensional simulations. J. Comput. Phys..

[115] Sethian J.A. (1999). Level Sets Methods and Fast Marching Methods.

[116] Sethian J.A. (2001). Evolution, implementation, and application of level set and fast marching methods for advancing fronts. J. Comput. Phys..

[117] Chopp D.L. (2001). Some improvements on the fast marching method. SIAM J. Sci. Comput..

[118] Zhao H.K. (2005). A fast sweeping method for Eikonal equations. Math. Comput..

[119] Gremaud P.A., Kuster C.M. (2006). Computational study of fast methods for the Eikonal equation. SIAM J. Sci. Comput..

[120] Yatziv L., Bartesaghi A., Sapiro G. (2006). O (N) implementation of the fast marching algorithm. J. Comput. Phys..

[121] Hassouna M.S., Farag A.A. (2007). Multistencils fast marching methods: A highly accurate solution to the Eikonal Equation on cartesian domains. IEEE Trans. Pattern Anal. Mach. Intell..

[122] IntelliSuite Software Brochure. www.intellisense.com.

[123] Ferrando N., Gosalvez M.A., Cerda J., Gadea R., Sato K. (2011). Octree-based, GPU implementation of a continuous cellular automaton for the simulation of complex, evolving surfaces. Comput. Phys. Commun..

[124] Chen J., Li J.H., Guo W. (2013). Improved parallel simulation of silicon anisotropic etching based on GPU. J. Comput. Appl..

